# Supplementary material for: Risk factors for Overweight/Obesity among people living with HIV on antiretroviral therapy: An ambidirectional cohort study at a tertiary health facility in Zambia
Source: PLoS One. 2025 Sep 8;20(9):e0330777. doi: 10.1371/journal.pone.0330777 (PMC12416660; doi:10.1371/journal.pone.0330777)
Supplement: S3 File — (DOCX) [file pone.0330777.s003.docx]

| Supplementary Table 1. End-of-Follow-up Demographic and Clinical Characteristics of Participants (N = 249) | |
| --- | --- |
| Variable | **End of Follow-up** |
| Follow-up duration, *months (IQR)* | 43 (42, 44) |
| Age, years (IQR) | 47 (40, 54) |
| Sex, n (%) |  |
| Male | 101 (40.6) |
| Female | 148 (59.4) |
| Marital status, n (%) |  |
| Married | 120 (48.2) |
| Unmarried | 129 (51.8) |
| Education level, n (%) |  |
| No formal schooling | 2 (0.8) |
| Primary | 48 (19.3) |
| Secondary | 152 (61.0) |
| Tertiary | 47 (18.9) |
| Work status, n (%) |  |
| Government employee | 19 (7.6) |
| Non-government | 47 (18.9) |
| Self-employed | 100 (40.2) |
| Unemployed | 83 (33.3) |
| Smoking status, n (%) |  |
| No | 229 (92.0) |
| Yes | 20 (8.0) |
| Alcohol use, n (%) |  |
| No | 182 (73.1) |
| Yes | 67 (26.9) |
| Systolic BP, mmHg (IQR) | 120 (110, 131) |
| Diastolic BP, mmHg (IQR) | 75.7 (70.7, 82.7) |
| Weight, kg (IQR) | 60.5 (52, 67) |
| Height, cm (IQR) | 165 (160, 171) |
| Body mass index, n (%) |  |
| Underweight | 43 (17.3) |
| Normal | 162 (65.1) |
| Overweight | 39 (15.7) |
| Obese | 5 (2.0) |
| ART regimen, n (%) |  |
| INSTI (DTG) | 249 (100) |
| Baseline NRTI, n (%) |  |
| ABC/3TC | 1 (0.4) |
| AZT/3TC | 22 (8.8) |
| TDF/3TC | 207 (83.1) |
| TAF/3TC | 19 (7.6) |
| Duration on ART, months (IQR) | 155 (97, 189) |
| Current duration on DTG based regimen, months (IQR) | 23 (19, 40) |
| Viral load, copies/mL | 0 (0, 30) |
| CD4 count, cells/µL (IQR) | 527 (387, 720) |
| Waist circumference, cm (IQR) | 77 (72, 85) |
| Hip circumference, cm (IQR) | 94 (89, 99) |
| LDL cholesterol, mmol/L (IQR) | 2.0 (1.4, 2.4) |
| Total cholesterol, mmol/L (IQR) | 3.4 (2.5, 3.9) |
| HDL cholesterol, mmol/L (IQR) | 0.9 (0.8, 1.2) |
| Physically active, n (%) |  |
| No | 101 (40.6) |
| Yes | 148 (59.4) |
| Note: Values are expressed as median (IQR) for continuous variables and n (%) for categorical variables. Abbreviations: BP = Blood Pressure; ART = Antiretroviral Therapy; NNRTI = Non-Nucleoside Reverse Transcriptase Inhibitor; PI = Protease Inhibitor; INSTI = Integrase Strand Transfer Inhibitor; NRTI = Nucleoside Reverse Transcriptase Inhibitor; EFV = Efavirenz; NVP = Nevirapine; LPV/r = Lopinavir/ritonavir; ATV/r = Atazanavir/ritonavir; DTG = Dolutegravir; ABC = Abacavir; 3TC = Lamivudine; AZT = Zidovudine; TDF = Tenofovir Disoproxil Fumarate; TAF = Tenofovir Alafenamide; LDL = Low-Density Lipoprotein; HDL = High-Density Lipoprotein ; IQR=Interquartile range | |
