# Supplementary material for: Risk factors for Overweight/Obesity among people living with HIV on antiretroviral therapy: An ambidirectional cohort study at a tertiary health facility in Zambia
Source: PLoS One. 2025 Sep 8;20(9):e0330777. doi: 10.1371/journal.pone.0330777 (PMC12416660; doi:10.1371/journal.pone.0330777)
Supplement: Attachment — Submitted filename: response to review.docx [file pone.0330777.s006.docx]

23/07/2025

PLOS ONE Journal

Dear Editor,

**Ref: Submission of a revised research article for peer review and publication consideration**

Reference to the above-mentioned subject. I am writing to submit a revised original research article titled " Risk Factors for Overweight/Obesity among People Living with HIV on Antiretroviral Therapy: A Cohort Study at a Tertiary Health Facility in Zambia.

We would like to thank the reviewers for taking the time to make suggestions that have improved our manuscript. We have revised the manuscript and addressed all concerns and suggestions. We now hope the current manuscript is acceptable for publication. Below are the point-by-point responses to all comments and suggestions.

Journal Requirements:

If the reviewer comments include a recommendation to cite specific previously published works, please review and evaluate these publications to determine whether they are relevant and should be cited. There is no requirement to cite these works unless the editor has indicated otherwise.

**Response**: thank you, noted.

Please review your reference list to ensure that it is complete and correct. If you have cited papers that have been retracted, please include the rationale for doing so in the manuscript text, or remove these references and replace them with relevant current references. Any changes to the reference list should be mentioned in the rebuttal letter that accompanies your revised manuscript. If you need to cite a retracted article, indicate the article’s retracted status in the References list and also include a citation and full reference for the retraction notice.

**Response:** thank you, all reviewed .

Additional Editor Comments:

Clarifications needed before final approval to publish
While the Author has addressed most queries given in the first rebuttal, it is still unclear with regards to:
1. This appears to be a retrospective cohort study given that data was collected at start and end point.

**Response:** Thank you for your observation. Indeed, there is a retrospective element to the study. Initial data were collected approximately three years ago, after which the same cohort was recalled, and key study variables were physically collected. Given that some data—such as ART regimen and duration on ART—were obtained from medical records (SmartCare), while other data—such as weight, height, and lipid profiles—were collected directly from participants, we believe this qualifies as an ambidirectional cohort study, which takes into account the retrospective and prospective nature of the data. The manuscript has been revised accordingly.

Most ART providing health facilities prescribe 3 monthly ART and clients have to return to the facility every three months for refill. Why did the authors not collect data at least every 6 months for the 3–4-year period and only collect data at baseline and end point?.

**Response:** Thank you very much for your concern. While we would have preferred to collect data at six-month intervals as suggested, limited funding constrained us to only two data collection points. In our setting, medical records often have significant gaps, and without a properly designed study, there is a high risk of underpowered results and substantial missing data. Hence we could only recall the participants after securing research funds. Nevertheless, we have acknowledged this limitation in the Discussion section of the manuscript. “The study has some strengths and weaknesses. The study has included only a single follow-up period, making it impossible to determine the exact point when individuals met the criteria for overweight or obesity”

2. The total follow-up time for each participants appears to be 3-4 years (between 2019/2020 to 2022-2023), and each participant was followed only once, meaning you collected data from each participant at baseline and at the end of data collection only. We know that weight changes rapidly over short periods of time, and many factors explain these changes. Making conclusions about changes in weight over a four-year period may not be very accurate and liable to a lot of biases and confounding.

**Response: thank you very much.** We have revised and modified the discussion and conclusion of this manuscript to be more cautious study.

3. Authors mention in the manuscript that they excluded 63 participants after enrolment due to death, pregnancy, loss to follow-up among others. Please note that in a cohort study, once people are enrolled into the study, they contribute some person time to the follow-up and so should not be excluded especially in the baseline and survival analysis. At what point did these people fall out of the study and how much follow-up time did they contribute to the study. Did any of them have the outcome of interest at the time of dropping out of the study?

**Response:** Thank you for the observation. Given the study design we used where we had only one follow-up period, we were unable to conduct survival analysis. We did not collect data on the exact time each of the events happened for all the excluded participants; our interest was to analyze data for all the participants who had a measurement on the outcome of interest at follow-up period. Therefore, we had to exclude anyone without the measurement on the outcome of interest. We have included a recommendation in the conclusion “. Longitudinal studies with serial measurements are needed to clarify the impact and timing of weight gain from integrase inhibitors in this population”

4. Table one should not have the follow-up data. It should only contain baseline demographics and should be on 312 participants that were enrolled into the study. Table one still has empty cells in the follow-up section. This is not acceptable.

**Response:** thank you very much. we have redone Table to only show the baseline characteristics and a supplementary for endeline characteristics of the participant. Regarding including 312 participants; our design in another way is a pre-post study design and our aim was seeing what outcome each participant will end up having at follow-up given the baseline characteristics. Therefore, excluded everyone without the measurement on the outcome of interest (We did not collect data on the exact time each of the events happened for all the excluded participants).

We have revised the manuscript and addressed all concerns raised. We want to thank you all again for the tremendous work and time that you committed to reviewing and correcting  our work. Our manuscript is much improved, and we are very grateful.

Please address all correspondence to [lukundosiame23@gmail.com](mailto:lukundosiame23@gmail.com) . We look forward to hearing from you at your earliest convenience.

Please do not hesitate to contact me should you have further questions.

Yours sincerely,

Dr. Lukundo Siame, Bsc., MBcHB.

Junior Residence Medical Livingstone University Teaching Hospital
